# Effects of repeated-sprint training on intermittent aerobic endurance, anaerobic performance, and repeated-sprint ability in female collegiate basketball players

**DOI:** 10.3389/fphys.2026.1884648

**Published:** 2026-10-13

**Authors:** Jing An, Lin Zhou, Zijiu Zhang, Hao Yu, Chao Wei

**Affiliations:** 1 School of Physical Education, Shandong University, Jinan, Shandong, China; 2 Beijing Sport University, Beijing, China; 3 Shandong Sport University, Jinan, China

**Keywords:** athletic conditioning, high-intensity interval training, intermittent exercise, sprint performance, team sports

## Abstract

**Objective:**

This study examined the effects of a 6-week repeated-sprint training (RST) intervention on intermittent aerobic endurance, anaerobic power, and repeated-sprint ability (RSA) in female collegiate basketball players compared with duration-matched continuous running.

**Methods:**

In this assessor-blinded, parallel-group randomized controlled trial, 24 female collegiate basketball players were randomly allocated in a 1:1 ratio to a repeated-sprint ability group (RSAG; *n* = 12) or a duration-matched continuous-running control group (CG; *n* = 12). Both groups continued their regular basketball training. In addition, the RSAG completed two RST sessions per week for 6 weeks, consisting of 2–3 sets of 6–10 repetitions of 30-m (2 × 15-m) shuttle sprints with an approximate work-to-rest ratio of 1:4, whereas the CG completed continuous running matched to the elapsed duration of the corresponding RSAG sessions. Pre- and post-intervention assessments included the Yo-Yo Intermittent Recovery Test Level 1 (Yo-Yo IR1), the 30-s Wingate Anaerobic Test, and a 6 × 30-m shuttle-sprint test.

**Results:**

Significant Group × Time interactions were observed for Yo-Yo IR1, peak power (PP), mean power (MP), and fatigue index (FI). After 6 weeks, the RSAG showed greater improvements than the CG in Yo-Yo IR1 (*p* < 0.001, *d* = 1.202), PP (*p* < 0.001, *d* = 0.855), MP (*p* < 0.001, *d* = 1.004), and FI (*p* < 0.001, *d* = 1.635). Both groups showed significant pre- to post-intervention improvements in RSA-best, whereas no significant Group × Time interaction was observed for RSA-best or the individual sprint-time outcomes.

**Conclusion:**

In this sample of female collegiate basketball players and under the present duration-matched training conditions, RST combined with regular basketball training was associated with greater improvements in intermittent aerobic endurance, anaerobic power, and fatigue resistance than continuous running. However, because internal training load was not quantified, the observed between-group differences cannot be attributed solely to training modality or interpreted as evidence that RST is superior under equivalent physiological-load conditions.

## 1 Introduction

Basketball is characterized by frequent high-intensity intermittent actions, including sprinting, jumping, shuffling, and rapid changes of direction, interspersed with brief periods of low-intensity recovery (Gottlieb et al., 2021). These demands place substantial stress on both aerobic and anaerobic energy systems (Fort-Vanmeerhaeghe et al., 2016). A well-developed aerobic system helps sustain performance across a game and supports recovery between high-intensity efforts, whereas anaerobic capacity is essential for explosive actions such as maximal sprints and vertical jumps (Naser et al., 2017). Therefore, the ability to perform repeated sprints with minimal performance decrement, commonly termed repeated-sprint ability (RSA), is considered an important performance component in many team sports, including basketball (Buchheit et al., 2010).

To meet these multifaceted physiological demands, coaches and sport scientists continue to seek efficient conditioning methods. High-intensity interval training (HIIT) has attracted considerable attention because it can induce meaningful physiological adaptations within a relatively short training time (Hostrup and Bangsbo, 2023; Yuan et al., 2024). Repeated-sprint training (RST), a specific form of HIIT, involves maximal all-out sprints of short duration (<10 s) separated by brief recovery periods (<60 s) (Faiss et al., 2013a). This training mode is intended to target the neuromuscular and metabolic factors underlying RSA (Tønnessen et al., 2011). Previous studies have shown that RST can improve anaerobic performance through adaptations such as enhanced phosphocreatine resynthesis, glycolytic enzyme activity, and muscle buffering capacity (Faiss et al., 2013b). RST may also improve aerobic fitness by accelerating oxygen uptake kinetics, promoting mitochondrial biogenesis, and increasing oxidative enzyme activity (Bailey et al., 2009; Wang et al., 2025). These combined adaptations make RST a relevant conditioning strategy for intermittent sports such as basketball.

Despite growing evidence supporting RST, much of the existing research has focused on male athletes in sports such as soccer (Soares-Caldeira et al., 2014; Zhu et al., 2025) and rugby (Galvin et al., 2013). Evidence remains limited in female athletes, particularly in collegiate basketball. Female athletes may show neuromuscular and physiological responses to training that differ from those of male athletes, which supports the need for sex-specific research (Cao et al., 2025). Although some HIIT protocols have been shown to improve performance in female soccer players (Arazi et al., 2017) and basketball players (Fang and Jiang, 2024), few studies have evaluated RST specifically in female collegiate basketball players or compared its effects with more traditional conditioning methods under time-matched conditions.

Therefore, this study investigated the effects of a 6-week RST program on intermittent aerobic endurance, anaerobic power, and RSA in female collegiate basketball players. In contrast to previous HIIT and sprint-interval training studies in female basketball players, the present study (a) employed a basketball-specific repeated-sprint shuttle protocol rather than generic interval running, (b) used continuous running matched to the duration of the RST sessions as the comparison condition, allowing the two training modes to be compared under an equivalent time commitment, and (c) concurrently assessed intermittent aerobic endurance (Yo-Yo Intermittent Recovery Test Level 1; Yo-Yo IR1), anaerobic power (Wingate test), and RSA within the same cohort of female collegiate basketball players, thereby providing a more comprehensive evaluation of RST in this under-researched population. We hypothesized that, compared with a duration-matched continuous aerobic training program, RST would elicit greater improvements in the measured performance outcomes.

## 2 Methods

### 2.1 Participants

An *a priori* power analysis was conducted using G*Power software (version 3.1.9.7; Heinrich Heine University, Düsseldorf, Germany) to determine the required sample size. Based on previous studies examining the effects of high-intensity interval training in basketball players, a medium-to-large effect size (*f* = 0.30) was anticipated. For a repeated-measures analysis of variance assessing the Group × Time interaction, the statistical parameters were set at α = 0.05, power (1 − β) = 0.80, two groups, and two measurement occasions (pre- and post-intervention). The analysis indicated that a minimum total sample of 24 participants was required to detect an interaction effect of the specified magnitude.

To account for an anticipated attrition rate of approximately 15%, 28 female collegiate basketball players from the Shandong University varsity team were assessed for eligibility. All players had at least 5 years of competitive basketball training experience. The inclusion criteria were as follows: (1) no history of lower-extremity musculoskeletal injury within the preceding 6 months; (2) no use of ergogenic aids, anabolic agents, or other performance-enhancing medications during the study; and (3) willingness and availability to participate in all scheduled training and testing sessions. Four players were excluded before randomization because of scheduling conflicts or minor injuries unrelated to the intervention; these players were not assigned to either study group. The remaining 24 participants completed the baseline assessments and proceeded to randomization.

After eligibility had been confirmed and baseline assessments had been completed, the 24 participants were randomly allocated in a 1:1 ratio to the repeated-sprint ability group (RSAG; *n* = 12) or the control group (CG; *n* = 12) (**Figure 1**). The allocation sequence was generated from a computer-generated random-number table created in Microsoft Excel (Microsoft Corporation, Redmond, WA, USA) by an independent researcher who was not involved in participant recruitment, intervention delivery, or outcome assessment. A fixed 1:1 allocation ratio was used. Neither permuted-block randomization nor stratified randomization based on playing position or baseline performance was applied.

**Figure 1:**
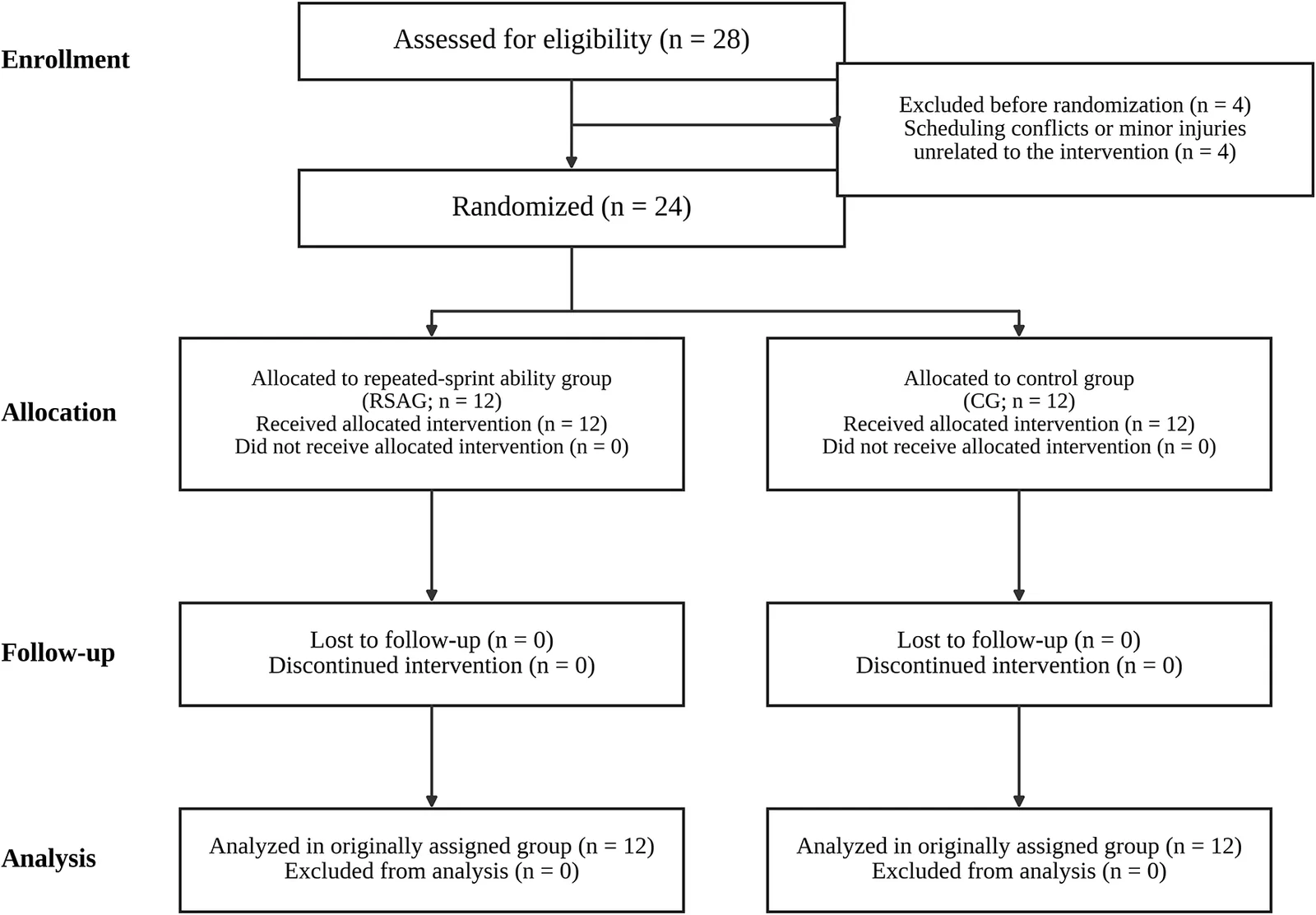
CONSORT flow diagram of participant recruitment, allocation, follow-up, and analysis.

Allocation concealment was maintained using sequentially numbered, opaque, sealed envelopes prepared and retained by the independent researcher. After each participant had completed the baseline assessments, the independent researcher opened the next envelope in numerical order and communicated the assignment only to the researcher responsible for supervising the intervention. Consequently, the researchers responsible for participant recruitment and baseline testing had no access to the allocation sequence and could not foresee the upcoming group assignment.

Because the two conditioning protocols were readily distinguishable, participants and the researchers supervising the training sessions could not be blinded to group allocation. However, the investigators responsible for the outcome assessments were not involved in sequence generation or allocation and remained blinded to group assignment during the post-intervention assessments. Participants were instructed not to disclose their group allocation to the assessors during testing.

Baseline characteristics of the randomized participants are summarized in **Table 1**. All participants met the eligibility criterion of at least 5 years of competitive basketball experience. Playing position was not prospectively recorded in the study database and therefore could not be summarized by randomized group or used for stratified randomization. Written informed consent was obtained from all participants before participation. The study protocol was approved by the Ethics Committee of Shandong University (No. IRB-SBMS-SDU (L) 2026-01-3) and was conducted in accordance with the Declaration of Helsinki. The study was not registered in a clinical trial registry because it involved a non-clinical physical-training intervention in healthy collegiate athletes.

**Table 1:** Baseline characteristics of the randomized participants.

Characteristic	RSAG (n = 12)	CG (n = 12)	P value	Absolute SMD
Age (years)	20.68 ± 0.69	20.42 ± 0.71	0.362	0.38
Height (cm)	171.57 ± 5.22	172.52 ± 4.11	0.626	0.20
Body mass (kg)	66.38 ± 3.77	65.80 ± 4.20	0.728	0.14
Body fat (%)	21.34 ± 3.38	21.51 ± 2.18	0.887	0.06
Competitive basketball experience ≥5 years, n (%)	12 (100)	12 (100)	—	—
Prescribed regular team training (min/week)	270	270	—	—

Values are presented as mean ± SD or n (%), as appropriate. RSAG, repeated-sprint ability group; CG, control group; SMD, standardized mean difference. The reported weekly training volume refers to the common prescribed regular basketball-training schedule of three 90-min sessions per week and does not represent individualized internal training load. All participants met the eligibility criterion of at least 5 years of competitive basketball experience; however, exact years of training experience were not prospectively recorded. Playing position, menstrual-cycle status, cycle phase at testing, and hormonal contraceptive use were not prospectively recorded and therefore could not be summarized by group.

### 2.2 Experimental design

An assessor-blinded, parallel-group randomized controlled trial with pre- and post-intervention assessments was conducted. The 6-week intervention was conducted during the pre-season phase to minimize the potential confounding effects of competitive fatigue. During the intervention, both groups maintained their regular technical and tactical basketball training, which consisted of three 90-min sessions per week. The conditioning intervention was performed immediately after the regular basketball-training component. The elapsed duration of each CG conditioning session was matched to that of the corresponding RSAG session, including work and recovery periods.

### 2.3 Training protocols

Both groups continued their regular technical and tactical basketball training throughout the 6-week intervention. Regular basketball practice was performed three times per week for 90 min per session, corresponding to 270 min of team training per week and 1,620 min over the entire intervention period. In addition, both groups completed two conditioning sessions per week for 6 weeks, resulting in 12 prescribed conditioning sessions. The detailed training progression for both groups is presented in **Table 2**.

**Table 2:** Detailed training progression for the repeated sprint ability group (RSAG) and control group (CG) over the 6-week intervention.

Weeks	Group	Training prescription	Repetitions per session	Distance per session	Weekly intervention volume	Intensity	Recovery
1–2	RSAG	2 sets × 6 × 30-m shuttle sprints	12	360 m	720 m/week	Maximal voluntary effort	24 s between repetitions; 3 min between sets
1–2	CG	Continuous court running	—	—	2 duration-matched sessions/week	65%–70% HRmax	Continuous
3–4	RSAG	2 sets × 8 × 30-m shuttle sprints	16	480 m	960 m/week	Maximal voluntary effort	24 s between repetitions; 3 min between sets
3–4	CG	Continuous court running	—	—	2 duration-matched sessions/week	70%–75% HRmax	Continuous
5–6	RSAG	3 sets × 10 × 30-m shuttle sprints	30	900 m	1,800 m/week	Maximal voluntary effort	24 s between repetitions; 3 min between sets
5–6	CG	Continuous court running	—	—	2 duration-matched sessions/week	70%–75% HRmax	Continuous

RSAG, repeated-sprint ability group; CG, control group; HRmax, maximum heart rate. Each 30-m repetition consisted of a 15-m outward sprint, a 180°change of direction, and a 15-m return sprint. The RSAG completed a prescribed cumulative sprint distance of 6,960 m across the 6-week intervention. Both groups also completed 270 min of regular basketball training per week.

Participants in the RSAG performed maximal-effort 30-m shuttle sprints, with each repetition consisting of a 15-m sprint, a 180° change of direction, and a 15-m return sprint. Each sprint lasted approximately 6 s and was followed by 24 s of passive recovery, corresponding to an approximate work-to-rest ratio of 1:4. A 3-min passive recovery period was provided between sets.

During weeks 1–2, participants completed 2 sets of 6 repetitions, corresponding to 12 shuttle sprints and a total sprint distance of 360 m per session, or 720 m per week. During weeks 3–4, the volume was increased to 2 sets of 8 repetitions, corresponding to 16 shuttle sprints and 480 m per session, or 960 m per week. During weeks 5–6, participants completed 3 sets of 10 repetitions, corresponding to 30 shuttle sprints and 900 m per session, or 1,800 m per week. Across the 12 conditioning sessions, each participant in the RSAG was prescribed a total of 232 shuttle sprints and a cumulative sprint distance of 6,960 m.

The progression was implemented by increasing the number of repetitions during the second training phase and subsequently increasing both the number of sets and repetitions during the final phase, while sprint intensity and recovery intervals were kept constant. The lower initial volume was intended to limit excessive neuromuscular and metabolic fatigue during the early phase of exposure to maximal shuttle sprinting. The subsequent increases in sprint volume were designed to progressively increase repeated phosphocreatine depletion, glycolytic demand, and the oxidative contribution required for recovery between sprints. The 24-s inter-repetition recovery allowed partial phosphocreatine resynthesis while maintaining cumulative metabolic stress, whereas the 3-min inter-set recovery was intended to preserve sprint quality during the subsequent set (Girard et al., 2011; Thurlow et al., 2023).

Participants in the CG performed continuous aerobic running on the basketball court. Running intensity was prescribed at 65%–70% of maximum heart rate (HRmax) during weeks 1–2 and 70%–75% HRmax during weeks 3–6. Maximum heart rate was estimated using an age-prediction equation. Individual session-level heart-rate data were not retained for internal-load analysis. The duration of each CG session was matched to the total elapsed duration of the corresponding RSAG session, including sprinting, inter-repetition recovery, and inter-set recovery. Because the two training modes differed substantially, the protocols were matched for elapsed conditioning time rather than running distance, mechanical work, or internal physiological load. Heart rate was used only to guide the prescribed running intensity in the CG; however, individual session-level heart-rate responses were not retained or analyzed as measures of internal training load, and comparable heart-rate data were not collected in the RSAG. Session rating of perceived exertion (session-RPE) and heart-rate-derived training impulse (TRIMP) were not collected in either group. Therefore, the interventions were matched for elapsed conditioning time but not for internal physiological load.

Attendance was recorded for every conditioning session. Intervention adherence was calculated for each participant as the number of fully completed conditioning sessions divided by the 12 prescribed sessions and multiplied by 100. A session was classified as completed when the participant performed the entire prescribed sprint protocol in the RSAG or the entire prescribed running duration in the CG.

### 2.4 Testing procedures

Pre-intervention assessments were conducted before the start of the 6-week intervention, and the same procedures were repeated following completion of the intervention. All assessments were performed between 09:00 and 11:00 to minimize potential circadian variation. Anthropometric measurements were obtained first. All three performance tests were subsequently completed during the same morning testing session in a fixed order: the Wingate Anaerobic Test, the Yo-Yo Intermittent Recovery Test Level 1 (Yo-Yo IR1), and the repeated-sprint ability (RSA) test. A standardized 30-min passive recovery interval was provided between the Wingate and Yo-Yo IR1 tests and between the Yo-Yo IR1 and RSA tests. The same test order and recovery intervals were applied during the pre- and post-intervention assessments.

Before the baseline assessments, all participants completed a separate familiarization session. During familiarization, the participants were introduced to and practiced the standardized test procedures, equipment, starting procedures, verbal instructions, and test-termination criteria. Familiarization data were not included in the statistical analysis.

The same assessor conducted all tests during the pre- and post-intervention assessments. The assessor was not involved in sequence generation or group allocation and remained blinded to group assignment during the post-intervention assessments. Standardized instructions and verbal encouragement were provided throughout the tests.

All court-based assessments were conducted on the same indoor synthetic-surface basketball court using identical distance markings and turning lines. Participants wore the same pair of basketball shoes during the pre- and post-intervention assessments. The same testing equipment and configurations were used at both assessment time points. Participants were instructed to refrain from strenuous physical activity for 24 h before each testing session.

Menstrual-cycle phase was neither controlled nor verified when scheduling the pre- and post-intervention performance assessments. Menstrual-cycle status and phase on each testing day, as well as hormonal contraceptive use, were not prospectively recorded; therefore, repeated assessments could not be standardized to the same menstrual-cycle phase.

#### 2.4.1 Anthropometrics

Height and body mass were measured using standard anthropometric procedures. Body fat percentage was assessed using a bioelectrical impedance analyzer (InBody 770; Biospace, Seoul, Korea).

#### 2.4.2 Intermittent aerobic endurance

The Yo-Yo Intermittent Recovery Test Level 1 (Yo-Yo IR1) was used to evaluate intermittent aerobic endurance. Participants performed 2 x 20 m shuttle runs at progressively increasing speeds dictated by audio signals, with a 10 s active recovery period between runs. The test was terminated when a participant failed to reach the finish line before the signal on two consecutive occasions. The total distance covered (m) was recorded as the performance outcome.

#### 2.4.3 Wingate-derived anaerobic performance

The Wingate Anaerobic Test (WAnT) was conducted on a cycle ergometer (Monark 894E, Monark Exercise AB, Vansbro, Sweden) to assess short-duration anaerobic performance. The resistance load was standardized at 7.5% of each participant’s body mass. Participants were instructed to pedal at maximal effort for 30 s. The primary outcomes were peak power (PP) and mean power (MP), which were interpreted as standardized cycle-ergometer measures of short-duration anaerobic power and capacity, respectively. These outcomes were not considered direct measures of basketball-specific explosive performance.

#### 2.4.4 Repeated sprint ability

RSA was assessed using a 6 x 30 m shuttle sprint test. Participants performed six maximal 30 m (2 x 15 m) sprints interspersed with 20 s of passive recovery. Sprint time was recorded automatically using a Witty electronic timing-gate system (Microgate Srl, Bolzano, Italy). The timing gates were positioned at the start/finish line, and the same gate height, placement, starting position, and timing configuration were used during the pre- and post-intervention assessments. The performance outcomes included best sprint time (RSA-best), defined as the fastest time among the six sprints, and fatigue index (FI). FI was calculated using the following formula:


$$\text{FI}=\frac{\text{Total Time}–({\text{RSA}}_{\text{best}}\text{x}6)}{{\text{RSA}}_{\text{best}}\text{x}6}\times 100\%$$

#### 2.4.5 Measurement reliability

No separate test–retest reliability session was conducted in the present cohort; therefore, study-specific intraclass correlation coefficients (ICCs) and coefficients of variation (CVs) were not calculated. Instead, published reliability data were used to support the selected assessments. The Yo-Yo IR1 has demonstrated good test–retest reliability, with a CV of 4.9% in adult male soccer players (Krustrup et al., 2003). For the 30-s Wingate test performed against a resistance equivalent to 7.5% of body mass, ICCs of 0.91 and 0.96 and relative standard errors of measurement of 5.5% and 2.6% have been reported for peak and mean power, respectively, in physically active women (Kavaliauskas and Phillips, 2016). In elite female field-hockey players, a comparable 6 × 40-m repeated shuttle-sprint test demonstrated an ICC of 0.92 and a CV of 0.76% for best sprint time, whereas the performance-decrement score demonstrated substantially lower reproducibility (CV = 29.0%) (Goods et al., 2022). In highly trained male field-hockey players, a 6 × 30-m straight-line repeated-sprint test demonstrated a typical error of 0.7% for total sprint time and 14.9% for percentage sprint decrement (Spencer et al., 2006). Collectively, these findings indicate that best and total sprint-time measures are generally more reproducible than fatigue- or decrement-based indices.

### 2.5 Statistical analysis

Statistical analyses were performed using JASP software (version 0.18.3; JASP Team, Amsterdam, the Netherlands). Data are presented as mean ± standard deviation (SD). Normality was assessed using the Shapiro–Wilk test. For each outcome, a 2 × 2 mixed-design analysis of variance (ANOVA) was performed, with Group (RSAG vs. CG) as the between-subject factor and Time (pre- vs. post-intervention) as the within-subject factor. Type III sums of squares were used. The main effects of Time and Group and the Group × Time interaction were evaluated. ANOVA results are reported as F statistics with the corresponding numerator and denominator degrees of freedom, p values, and partial eta squared (ηp^2^). Because the within-subject factor Time consisted of only two levels, the assumption of sphericity was inherently satisfied and no sphericity correction was required. To control the family-wise Type I error associated with the six sprint-specific outcomes (RSA-1 to RSA-6), Holm adjustment was additionally applied across the six Group × Time interaction p values.

When a significant Group × Time interaction was identified, Bonferroni-adjusted pairwise comparisons were performed to examine the simple pre–post effects within each group. Mean differences with 95% confidence intervals (CIs), adjusted p values, and Cohen’s d effect sizes were reported for these comparisons. Cohen’s d values were obtained from the JASP *post-hoc* pairwise comparisons and represent standardized pairwise mean differences; the within-group Cohen’s d values were used to describe the magnitude of pre–post changes, whereas partial ηp^2^ for the Group × Time interaction was used to quantify the magnitude of the differential intervention effect between groups. For sprint-time outcomes, a lower value represents better performance. Therefore, statistically significant changes in sprint time were not automatically interpreted as performance improvements. Statistical significance was set at p < 0.05. All randomized participants were analyzed in the groups to which they were allocated. Because no participant withdrew after randomization and no outcome data were missing, the intention-to-treat and per-protocol populations were identical; therefore, no imputation was required.

## 3 Results

Of the 28 players assessed for eligibility, four were excluded before randomization because of scheduling conflicts or minor injuries unrelated to the intervention. The remaining 24 participants were randomized to the RSAG (n = 12) or CG (n = 12). No participants were lost to follow-up, discontinued the intervention, or were excluded from the analysis. All participants completed all 12 prescribed conditioning sessions, corresponding to an intervention adherence rate of 100% in both groups. Thus, all 24 randomized participants were analyzed in their originally assigned groups. Baseline characteristics of the randomized participants are presented in **Table 1**. The two groups were descriptively comparable with respect to the available anthropometric characteristics. All participants met the eligibility criterion of at least 5 years of competitive basketball experience and followed the same prescribed regular team-training schedule of 270 min/week. Playing position was not available for group-level reporting.

The mixed-design ANOVA revealed significant Group × Time interactions for Yo-Yo IR1 (F(1, 22) = 14.838, p < 0.001, ηp^2^ = 0.403), PP (F(1, 22) = 42.620, p < 0.001, ηp^2^ = 0.660), MP (F(1, 22) = 37.968, p < 0.001, ηp^2^ = 0.633), and FI (F(1, 22) = 36.520, p < 0.001, ηp^2^ = 0.624). Significant main effects of Time were also observed for Yo-Yo IR1 (F(1, 22) = 67.223, p < 0.001, ηp^2^ = 0.753), PP (F(1, 22) = 69.559, p < 0.001, ηp^2^ = 0.760), MP (F(1, 22) = 50.589, p < 0.001, ηp^2^ = 0.697), and FI (F(1, 22) = 119.478, p < 0.001, ηp^2^ = 0.844). Bonferroni-adjusted *post-hoc* comparisons showed that Yo-Yo IR1 increased significantly in both the RSAG (mean change = 186.67 m, 95% CI: 123.17 to 250.16 m, p < 0.001, d = 1.202) and CG (mean change = 67.33 m, 95% CI: 3.84 to 130.83 m, p = 0.033, d = 0.433). PP increased significantly in the RSAG (mean change = 43.08 W, 95% CI: 31.21 to 54.96 W, p < 0.001, d = 0.855), whereas no significant pre–post change was observed in the CG (mean change = 5.25 W, 95% CI: −6.63 to 17.13 W, adjusted p = 1.000, d = 0.104). Similarly, MP increased significantly in the RSAG (mean change = 30.25 W, 95% CI: 20.91 to 39.59 W, p < 0.001, d = 1.004), but not in the CG (mean change = 2.17 W, 95% CI: −7.18 to 11.51 W, adjusted p = 1.000, d = 0.072). FI decreased significantly in both groups, with a larger change observed in the RSAG (mean change = −1.77 percentage points, 95% CI: −2.20 to −1.34, p < 0.001, d = 1.635) than in the CG (mean change = −0.51 percentage points, 95% CI: −0.94 to −0.08, p = 0.013, d = 0.471) (**Figure 2**; **Table 3**).

**Figure 2:**
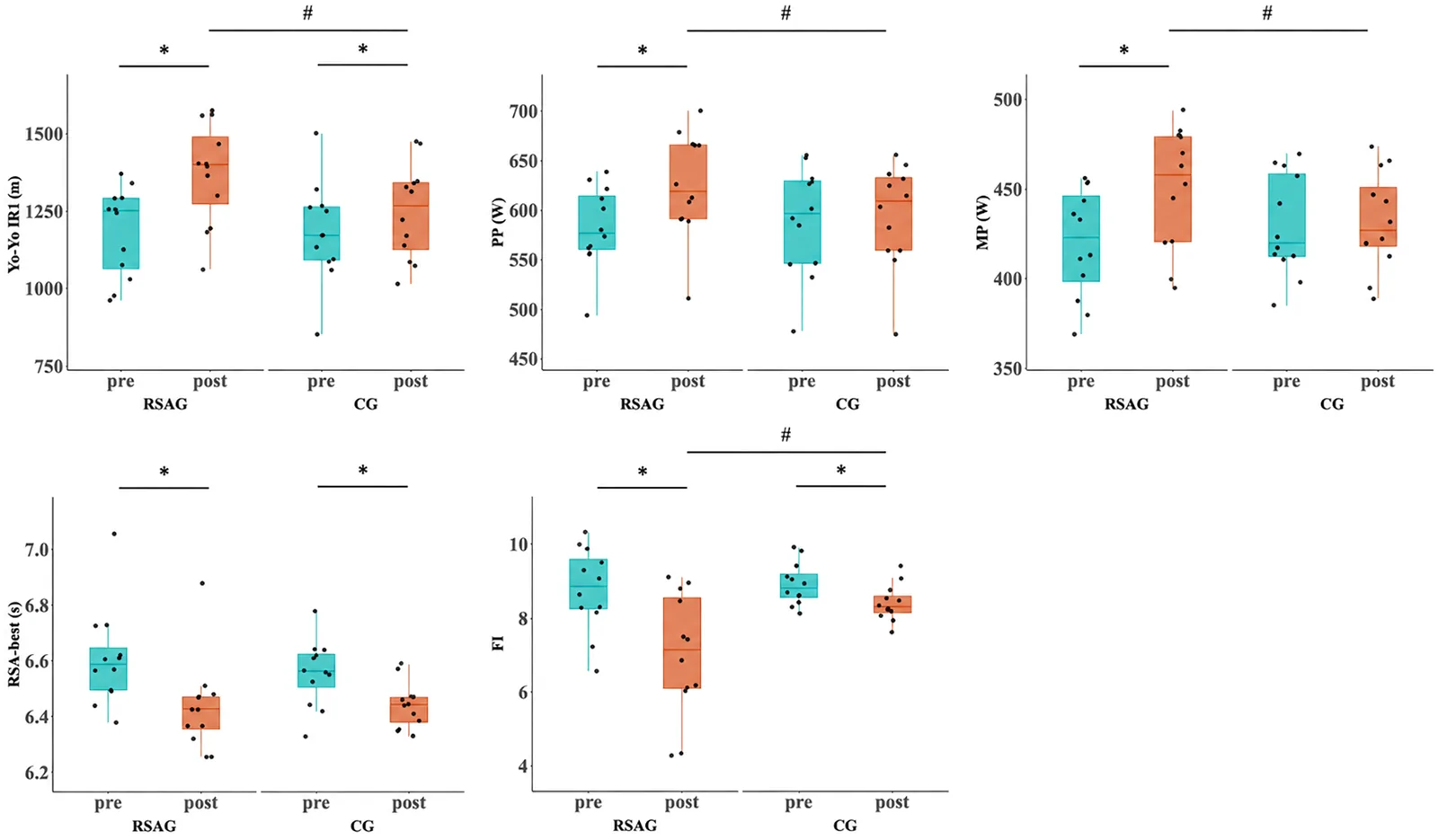
Changes in intermittent aerobic endurance (Yo-Yo IR1), anaerobic power (PP and MP), best repeated-sprint time (RSA-best), and fatigue index before and after the 6-week intervention in the repeated sprint ability group (RSAG) and control group (CG). Values are presented as mean ± SD. Group × Time interaction effect sizes are presented as partial eta squared (ηp^2^) within each panel. *p < 0.05 for the Bonferroni-adjusted within-group pre–post comparison. For RSA-best and FI, lower values indicate better performance.

**Table 3:** Main performance outcomes for the RSAG and CG before and after the 6-week training intervention.

Outcome	RSAG (n = 12)		CG (n = 12)		Time effect	Time × group interaction
	Pre	Post	Pre	Post	F(1, 22)/p/ηp^2^	F(1, 22)/p/ηp^2^
Yo-Yo IR1 (m)	1186.25 ± 144.19	1372.92 ± 163.10*	1181.75 ± 160.09	1249.08 ± 153.31*	67.223/<0.001/0.753	14.838/<0.001/0.403
PP (W)	582.75 ± 40.88	625.83 ± 52.87*	590.00 ± 54.55	595.25 ± 52.10	69.559/<0.001/0.760	42.620/<0.001/0.660
MP (W)	419.92 ± 30.48	450.17 ± 33.80*	429.83 ± 28.54	432.00 ± 27.29	50.589/<0.001/0.697	37.968/<0.001/0.633
RSA-best (s)	6.61 ± 0.18	6.44 ± 0.16*	6.56 ± 0.12	6.44 ± 0.08*	93.131/<0.001/0.809	3.389/0.079/0.133
FI (%)	8.78 ± 1.14	7.02 ± 1.68*	8.93 ± 0.57	8.42 ± 0.49*	119.478/<0.001/0.844	36.520/<0.001/0.624

Values are presented as mean ± SD. RSAG = Repeated Sprint Ability Group; CG = Control Group; ηp^2^ = partial eta squared. The final two columns report F(1, 22)/p/ηp^2^. * p < 0.05 for the Bonferroni-adjusted within-group pre–post comparison. Lower RSA-best and FI values indicate better performance.

For RSA-best, there was a significant main effect of Time (F(1, 22) = 93.131, p < 0.001, ηp^2^ = 0.809), whereas the Group × Time interaction did not reach statistical significance (F(1, 22) = 3.389, p = 0.079, ηp^2^ = 0.133). Bonferroni-adjusted comparisons showed significant reductions in RSA-best in both the RSAG (mean change = −0.172 s, 95% CI: −0.233 to −0.110 s, p < 0.001, d = 1.227) and CG (mean change = −0.117 s, 95% CI: −0.178 to −0.055 s, p < 0.001, d = 0.834), indicating faster best-sprint performance in both groups. However, the nonsignificant Group × Time interaction indicates that the magnitude of improvement did not differ statistically between groups. For the individual sprint times (RSA-1 to RSA-6), significant main effects of Time were observed for all six sprints (all p < 0.001). However, no significant Group × Time interactions were detected for RSA-1 (F(1, 22) < 0.001, p = 1.000, ηp^2^ < 0.001), RSA-2 (F(1, 22) = 1.180, p = 0.289, ηp^2^ = 0.051), RSA-3 (F(1, 22) = 0.572, p = 0.457, ηp^2^ = 0.025), RSA-4 (F(1, 22) = 0.854, p = 0.365, ηp^2^ = 0.037), RSA-5 (F(1, 22) = 0.374, p = 0.547, ηp^2^ = 0.017), or RSA-6 (F(1, 22) = 0.325, p = 0.574, ηp^2^ = 0.015). To account for multiplicity across the six sprint-specific Group × Time interaction tests, Holm adjustment was applied; all adjusted p values were 1.000, leaving the interpretation unchanged.

Importantly, the individual sprint times generally increased from pre- to post-intervention in both groups, indicating slower rather than faster sprint performance. Therefore, these statistically significant Time effects should be interpreted as changes in sprint performance rather than improvements. The absence of significant Group × Time interactions further indicates that this temporal pattern did not differ between the RSAG and CG (**Figure 3**; **Table 4**).

**Figure 3:**
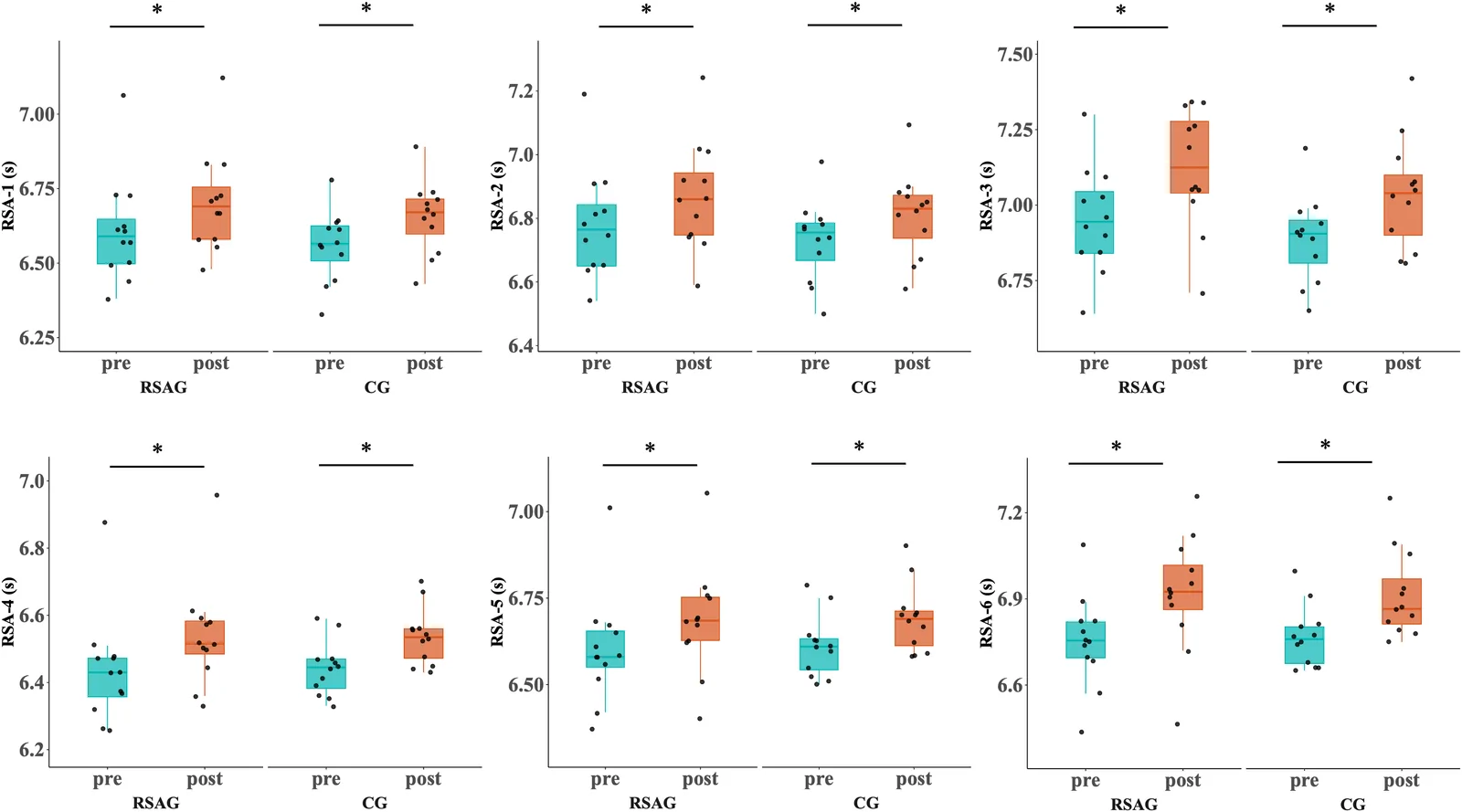
Changes in individual sprint times (RSA-1 to RSA-6) during the repeated sprint ability test before and after the 6-week intervention in the repeated sprint ability group (RSAG) and control group (CG). Values are presented as mean ± SD. Group × Time interaction effect sizes are presented as partial eta squared (ηp^2^) within each panel. None of the Group × Time interactions remained statistically significant after correction for multiple comparisons. *p < 0.05 for the Bonferroni-adjusted within-group pre–post comparison. The asterisk indicates a statistically significant pre–post difference and does not necessarily indicate performance improvement. For sprint-time outcomes, lower values indicate better performance; therefore, an increase in sprint time represents slower performance.

**Table 4:** Sprint-specific outcomes during the repeated-sprint ability test for the RSAG and CG before and after the 6-week training intervention.

Outcome	RSAG (n = 12)		CG (n = 12)		Time effect	Time × group interaction
	Pre	Post	Pre	Post	F(1, 22)/p/ηp^2^	F(1, 22)/p/ηp^2^
RSA-1 (s)	6.61 ± 0.18	6.71 ± 0.17*	6.56 ± 0.12	6.65 ± 0.12*	1480.160/<0.001/0.985	<0.001/1.000/<0.001
RSA-2 (s)	6.78 ± 0.17	6.87 ± 0.17*	6.73 ± 0.13	6.81 ± 0.13*	481.592/<0.001/0.956	1.180/0.289/0.051
RSA-3 (s)	6.95 ± 0.17	7.12 ± 0.20*	6.89 ± 0.14	7.04 ± 0.18*	135.748/<0.001/0.861	0.572/0.457/0.025
RSA-4 (s)	6.44 ± 0.16	6.54 ± 0.16*	6.44 ± 0.08	6.54 ± 0.08*	979.174/<0.001/0.978	0.854/0.365/0.037
RSA-5 (s)	6.60 ± 0.16	6.69 ± 0.16*	6.61 ± 0.09	6.69 ± 0.10*	390.562/<0.001/0.947	0.374/0.547/0.017
RSA-6 (s)	6.75 ± 0.16	6.92 ± 0.20*	6.77 ± 0.11	6.91 ± 0.15*	103.685/<0.001/0.825	0.325/0.574/0.015

Values are presented as mean ± SD. RSAG = Repeated Sprint Ability Group; CG = Control Group; ηp^2^ = partial eta squared. The final two columns report F(1, 22)/p/ηp^2^. * p < 0.05 for the Bonferroni-adjusted within-group pre–post comparison. Asterisks indicate significant pre–post differences, not necessarily improvement. Lower sprint times indicate better performance; increases indicate slower performance. Holm adjustment was applied across the six Group × Time interaction tests; all adjusted p values were 1.000.

## 4 Discussion

The primary aim of this study was to investigate the effects of a 6-week RST program on intermittent aerobic endurance, Wingate-derived anaerobic performance, and RSA in female collegiate basketball players. The main finding was that both the RST and continuous-training groups improved selected physical performance variables, but the RSAG showed greater gains in intermittent endurance, anaerobic power, and fatigue resistance. These findings suggest that, under the duration-matched conditions used in this study, RST may be a time-efficient strategy for addressing the combined aerobic and anaerobic demands of female basketball, although both approaches contributed to some performance changes. However, because internal training load was not quantified, the between-group differences cannot be attributed solely to training mode or interpreted as evidence of superiority under equivalent physiological loads.

Matching elapsed conditioning time did not ensure equivalence of internal training load between the two groups. Internal training load represents the individual psychophysiological response to an externally prescribed training stimulus, whereas external training load describes the work completed by the athlete (Bourdon et al., 2017). In the present study, the RSAG performed maximal shuttle sprints involving repeated accelerations, decelerations, changes of direction, and brief passive recovery periods, whereas the CG performed submaximal continuous running. Consequently, the two protocols may have imposed different cardiovascular, metabolic, neuromuscular, and perceptual demands despite having the same elapsed duration.

Because session-level heart-rate responses, session-RPE, and TRIMP were not available, it was not possible to determine whether the greater improvements in Yo-Yo IR1 performance, peak power, mean power, and fatigue index in the RSAG resulted from the intermittent sprint modality itself, a greater or qualitatively different internal physiological load, or an interaction between training mode and load magnitude. Accordingly, the present findings should be interpreted as a comparison between the two complete conditioning programs as implemented, rather than as evidence that RST was superior to continuous running when the two modalities were matched for internal physiological load.

Future studies should combine continuous heart-rate monitoring with session-RPE and appropriate heart-rate-derived indices, together with external-load measures such as sprint volume, sprint-time decrement, and completed running distance. This multimethod approach would allow researchers to determine whether between-group adaptations remain different after internal load has been matched or statistically accounted for.

### 4.1 Intermittent aerobic endurance adaptations

A notable finding was the greater improvement in Yo-Yo IR1 performance in the RSAG than in the CG. This improvement is relevant because the work bouts during RST are performed predominantly under anaerobic conditions. The result is consistent with previous studies in team-sport athletes, including soccer and handball players, showing that high-intensity interval training can improve VO2max and intermittent endurance to an extent comparable to or greater than continuous endurance training (Belkadi et al., 2024; Bici and Kasa, 2025; Howard and Stavrianeas, 2017; Neves et al., 2022). For example, Belkadi et al. (2024) reported significant improvements in Yo-Yo IR1 performance after repeated-sprint exercise in handball players. The physiological basis for this crossover effect may involve the reliance on oxidative metabolism for phosphocreatine (PCr) resynthesis and intracellular lactate clearance during brief recovery periods (Tortu and Deliceoğlu, 2024). Consequently, the aerobic system is repeatedly taxed at high intensity to support recovery between sprints. This interpretation is consistent with Combes et al. (2018), who suggested that fluctuations in ATP demand during intermittent exercise may provide a stronger stimulus for mitochondrial biogenesis and oxidative enzyme activity than the steady-state demand of continuous running. Although the CG also improved Yo-Yo IR1 performance, the magnitude of improvement was smaller, indicating a larger response to RST under the present duration-matched design. However, because internal training load was not quantified, this difference cannot be attributed solely to training mode.

### 4.2 Wingate-derived anaerobic performance

The RSAG demonstrated greater increases in Wingate PP and MP than the CG. This outcome is consistent with the high-intensity nature of the RST protocol, which required repeated maximal-effort sprinting and imposed substantial neuromuscular and anaerobic metabolic demands. Improvements in peak and mean power following sprint-based training may be associated with enhanced neural drive, greater motor-unit recruitment, and adaptations in anaerobic glycolytic pathways, including changes in phosphofructokinase and lactate dehydrogenase activity (Linossier et al., 2004; Thurlow et al., 2023). In contrast, the submaximal continuous-running protocol may have provided a smaller stimulus for the development of short-duration anaerobic power.

The Wingate test has several methodological strengths. It provides a standardized and objective laboratory assessment of short-duration lower-limb anaerobic performance, and its resistance can be normalized to body mass. Published evidence in physically active women has demonstrated good test–retest reliability for 30-s Wingate peak and mean power, with ICCs of 0.91 and 0.96, respectively (Kavaliauskas and Phillips, 2016). The use of the same cycle ergometer, relative resistance, instructions, and testing procedures at the pre- and post-intervention assessments also reduced procedural variability in the present study.

Nevertheless, the Wingate test is not fully specific to basketball performance. It involves seated, cyclic pedaling on a stationary ergometer, whereas basketball requires weight-bearing accelerations and decelerations, multidirectional changes of direction, lateral movements, jumping, landing, and sport-specific technical actions. Consequently, improvements in Wingate PP and MP should be interpreted as improvements in cycle-ergometer-derived anaerobic power and capacity rather than as direct evidence of improved basketball-specific lower-limb explosiveness or match performance. The present findings therefore cannot establish whether the observed physiological adaptations transferred to vertical-jump performance, change-of-direction speed, basketball-specific sprinting, or competitive performance.

In addition, the greater improvements in PP and MP should not be attributed exclusively to the sprint modality because internal training load was not quantified. The possibility that the RSAG experienced a greater or qualitatively different metabolic and neuromuscular training dose cannot be excluded. Future studies should combine the Wingate test with basketball-relevant performance assessments, such as countermovement-jump height and power, short-distance sprint time, change-of-direction tests, and match-derived indicators of high-intensity activity. This combined approach would provide a more comprehensive evaluation of both general anaerobic adaptation and its transfer to basketball-specific performance.

### 4.3 Repeated-sprint ability and fatigue resistance

For RSA, both groups showed significant pre- to post-intervention changes in RSA-best, whereas the Time x Group interaction was not significant. This indicates that 6 weeks of RST did not produce a statistically superior improvement in maximal sprint performance compared with regular basketball training plus continuous running. This finding contrasts with studies reporting greater speed gains after RST (Negra et al., 2023; Thurlow et al., 2023), but it is consistent with evidence suggesting that maximal speed depends strongly on neural factors and individual characteristics that may require longer interventions or specific resistance training to change meaningfully (Douglas et al., 2021; Haugen et al., 2019). The principal adaptation elicited by RST in this study therefore appears to be improved fatigue resistance during repeated high-intensity efforts rather than superior maximal sprint velocity. This interpretation is consistent with Girard et al. (2011), who suggested that RSA improvements are mediated by several factors, including intramuscular buffering capacity and ion regulation. The ability to maintain sprint performance is important in basketball because decrements in speed during later stages of play may influence game outcomes.

### 4.4 Limitations and practical applications

Several limitations should be considered when interpreting the present findings. First, although the elapsed conditioning duration was matched between groups, internal training load was not quantified using session rating of perceived exertion (session-RPE), session-level heart-rate responses, time spent in heart-rate zones, or heart-rate-derived training impulse (TRIMP). Consequently, equivalence of the physiological training dose between groups could not be confirmed, and internal load could not be examined as a covariate or potential mediator of the observed adaptations. The between-group differences may therefore have reflected the training modality, the magnitude or nature of the internal physiological load, or an interaction between these factors. This limitation does not invalidate the randomized comparison between the two conditioning programs as implemented, but it precludes interpretation of the findings as evidence that RST is superior to continuous running under equivalent physiological-load conditions. Future studies should prospectively monitor both internal and external training loads and, where feasible, match or statistically account for internal load when comparing different conditioning modalities.

Second, the intervention was conducted during the pre-season while participants continued their regular technical and tactical basketball training. Although the groups were randomized and followed the same prescribed team-training schedule, individual exposure to basketball practice and technical–tactical training load was not strictly quantified. Differences in cumulative training exposure or fatigue may therefore have influenced individual responses to the conditioning interventions.

Third, menstrual-cycle phase was neither controlled nor verified during performance testing. Menstrual-cycle status, cycle phase on each testing day, and hormonal contraceptive use were not prospectively recorded. Consequently, the pre- and post-intervention assessments may have occurred during different menstrual-cycle phases, and unmeasured cycle-related physiological variation may have increased both within- and between-participant variability. Future studies involving female athletes should prospectively document menstrual-cycle characteristics and hormonal contraceptive use and, where feasible, conduct repeated assessments during comparable cycle phases.

Fourth, the Wingate, Yo-Yo IR1, and RSA tests were completed during the same morning testing session, and no study-specific test–retest reliability assessment was conducted. Although the testing order, 30-min inter-test recovery intervals, assessor, equipment, venue, court surface, and footwear were standardized across assessment time points, residual fatigue from preceding tests and measurement error cannot be completely excluded. This consideration may be particularly relevant to FI because fatigue- or decrement-based indices generally demonstrate lower reproducibility than best or total sprint-time measures. The FI findings should therefore be interpreted with appropriate caution, and future studies should consider conducting maximal anaerobic, intermittent-endurance, and repeated-sprint assessments on separate days and establishing protocol-specific reliability in the target population.

Fifth, although the Wingate test provides a standardized and reproducible assessment of cycle-ergometer-derived anaerobic performance, its movement pattern is not mechanically specific to basketball. Seated cycling does not reproduce the weight-bearing accelerations, decelerations, changes of direction, lateral movements, jumps, and landings required during basketball. Improvements in PP and MP therefore cannot be assumed to represent equivalent improvements in basketball-specific lower-limb explosiveness or match performance. Future studies should combine laboratory-based anaerobic testing with basketball-specific measures such as countermovement-jump performance, short-distance sprint time, change-of-direction speed, and match-derived indicators of high-intensity activity.

Sixth, this was a single-center study involving a relatively small sample from one university team. Although the sample size met the *a priori* power requirement, the precision of the effect estimates remains limited. In addition, playing position was not prospectively recorded, and neither block randomization nor stratification according to playing position or baseline performance was used. Chance imbalance in playing role or other measured and unmeasured prognostic characteristics therefore cannot be excluded, and position-specific differences in training responses could not be evaluated. Future studies should recruit larger multicenter samples, prospectively record playing position and training history, and consider stratified randomization or minimization based on playing position and key baseline performance variables. Finally, because all participants were female collegiate basketball players with at least 5 years of competitive training experience, the findings should be generalized cautiously to athletes of different sexes, ages, sports, or competition levels.

From a practical perspective, coaches may consider implementing the present RST protocol twice weekly for 6 weeks during the pre-season when the training objectives include intermittent aerobic endurance, Wingate-derived anaerobic power, and fatigue resistance. The progression used in this study consisted of 2 sets of 6 repetitions during weeks 1–2, 2 sets of 8 repetitions during weeks 3–4, and 3 sets of 10 repetitions during weeks 5–6. Each repetition comprised a maximal-effort 30-m shuttle sprint (2 × 15 m), with 24 s of passive recovery between repetitions and 3 min of passive recovery between sets.

Under these conditions, RST may be used to replace part of the continuous-running conditioning volume rather than serving as a universal replacement for all aerobic conditioning. However, coaches should not assume that protocols matched for elapsed duration impose equivalent physiological stress. Individual responses should be monitored using session-RPE, heart rate, sprint-time decrement, recovery status, and the broader weekly training load. The prescribed number of repetitions or sets should be reduced or progression delayed when excessive fatigue or impaired sprint quality is observed. Because playing position was not recorded or analyzed in the present study, no position-specific prescription can be derived from these findings; training progression should instead be individualized according to each athlete’s performance response, recovery capacity, and overall training exposure.

## 5 Conclusion

This assessor-blinded, parallel-group randomized controlled trial provides a direct duration-matched comparison of basketball-specific RST and continuous running in female collegiate basketball players while concurrently evaluating intermittent aerobic endurance, Wingate-derived anaerobic performance, and RSA. Under the present training conditions, the 6-week RST program was associated with greater improvements in Yo-Yo IR1 performance, Wingate PP and MP, and FI than continuous running, whereas the Group × Time interactions for RSA-best and the individual sprint-time outcomes were not significant. Because internal training load was not quantified, the observed differences may reflect training modality, physiological-load magnitude, or an interaction between these factors and should not be interpreted as evidence that RST is superior under equivalent physiological-load conditions. Future multicenter studies should include larger and position-stratified samples, prospective menstrual-cycle monitoring, internal-load measures, and basketball-specific outcomes such as vertical-jump performance, change-of-direction speed, and match-performance indicators.

## Data Availability

The original contributions presented in the study are included in the article/supplementary material. Further inquiries can be directed to the corresponding author.

## References

[B1] Arazi H. Keihaniyan A. EatemadyBoroujeni A. Oftade A. Takhsha S. Asadi A. . (2017). Effects of heart rate vs. speed-based high intensity interval training on aerobic and anaerobic capacity of female soccer players. Sports (Basel Switzerland) 5 (3), 57. doi: 10.3390/sports5030057 29910417 PMC5968964

[B2] Bailey S. J. Wilkerson D. P. Dimenna F. J. Jones A. M. (2009). Influence of repeated sprint training on pulmonary O2 uptake and muscle deoxygenation kinetics in humans. J. Appl. Physiol. (Bethesda Md. 1985) 106, 1875–1887. doi: 10.1152/japplphysiol.00144.2009 19342439

[B3] Belkadi A. Beboucha W. Benhammou S. Moussa M. Bouzoualegh M. Dairi A. (2025). Effects of concurrent in-season training on physiological functions required for top handball performance athletes. Sci. J. Sport Perform. 4 (1), 40–54. doi: 10.55860/JIXW8099

[B4] Bici P. A. Kasa A. (2025). Effects of high-intensity interval repeated sprint training with change of direction on aerobic and anaerobic capacity in 16-year-old male soccer players. Int. J. Phys. Education Sports Health. doi: 10.22271/kheljournal.2025.v12.i3c.3788

[B5] Bourdon P. C. Cardinale M. Murray A. Gastin P. Kellmann M. Varley M. C. . (2017). Monitoring athlete training loads: Consensus statement. Int. J. Sports Physiol. Perform. 12, S2-161–S162-170. doi: 10.1123/ijspp.2017-0208 28463642

[B6] Buchheit M. Mendez-Villanueva A. Delhomel G. Brughelli M. Ahmaidi S. (2010). Improving repeated sprint ability in young elite soccer players: Repeated shuttle sprints vs. explosive strength training. J. Strength Conditioning Res. 24, 2715–2722. doi: 10.1519/JSC.0b013e3181bf0223 20224449

[B7] Cao J. Lin J. Wang X. Wang Y. (2025). Effects of multiple-intensity SSIT pairing patterns on explosive power and skill performance in female basketball players. Front. Physiol. 16, 1635508. doi: 10.3389/fphys.2025.1635508 40988721 PMC12451168

[B8] Combes A. Dekerle J. Bougault V. Daussin F. (2018). Physiological comparison of intensity-controlled, isocaloric intermittent and continuous exercise†. Eur. J. Sport Sci. 18, 1368–1375. doi: 10.1080/17461391.2018.1491627 29975588

[B9] Douglas J. Ross A. Martin J. (2021). Maximal muscular power: Lessons from sprint cycling. Sports Med. - Open 7 (48). doi: 10.1186/s40798-021-00341-7 34268627 PMC8282832

[B10] Faiss R. Girard O. Millet G. P. (2013a). Advancing hypoxic training in team sports: From intermittent hypoxic training to repeated sprint training in hypoxia. Br. J. Sports Med. 7 (48), i45–i50. doi: 10.1136/bjsports-2013-092741 24282207 PMC3903143

[B11] Faiss R. Léger B. Vesin J. M. Fournier P. E. Eggel Y. Dériaz O. . (2013b). Significant molecular and systemic adaptations after repeated sprint training in hypoxia. PloS One 8, e56522. doi: 10.1371/journal.pone.0056522 23437154 PMC3577885

[B12] Fang K. Jiang H. (2024). Gender-specific effects of short sprint interval training on aerobic and anaerobic capacities in basketball players: A randomized controlled trial. J. Sports Sci. Med. 23, 8–16. doi: 10.52082/jssm.2024.8 38455442 PMC10915601

[B13] Fort-Vanmeerhaeghe A. Montalvo A. Latinjak A. Unnithan V. (2016). Physical characteristics of elite adolescent female basketball players and their relationship to match performance. J. Hum. Kinetics 53, 167–178. doi: 10.1515/hukin-2016-0020 28149421 PMC5260586

[B14] Galvin H. M. Cooke K. Sumners D. P. Mileva K. N. Bowtell J. L. (2013). Repeated sprint training in normobaric hypoxia. Br. J. Sports Med. 47 Suppl 1, i74–i79. doi: 10.1136/bjsports-2013-092826 24282212 PMC3903144

[B15] Girard O. Mendez-Villanueva A. Bishop D. (2011). Repeated-sprint ability - part I: Factors contributing to fatigue. Sports Med. 41, 673–694. doi: 10.2165/11590550-000000000-00000 21780851

[B16] Goods P. S. McKay A. K. Appleby B. Veli D. Peeling P. Jennings D. (2022). A repeated shuttle sprint test with female and male international field hockey players is reliable and associated with single sprint but not intermittent endurance performance. PloS One 17, e0271244. doi: 10.1371/journal.pone.0271244 35830427 PMC9278775

[B17] Gottlieb R. Shalom A. Calleja-Gonzalez J. (2021). Physiology of basketball - field tests. Review article. J. Hum. Kinetics 77, 159–167. doi: 10.2478/hukin-2021-0018 34168701 PMC8008295

[B18] Haugen T. Seiler S. Sandbakk Ø. Tønnessen E. (2019). The training and development of elite sprint performance: An integration of scientific and best practice literature. Sports Med. - Open 5, 44. doi: 10.1186/s40798-019-0221-0 31754845 PMC6872694

[B19] Hostrup M. Bangsbo J. (2023). Performance adaptations to intensified training in top-level football. Sports Med. (Auckland N.Z.) 53, 577–594. doi: 10.1007/s40279-022-01791-z 36380164 PMC9667002

[B20] Howard N. Stavrianeas S. (2017). In-season high-intensity interval training improves conditioning in high school soccer players. Int. J. Exercise Sci. 10, 713–720. doi: 10.70252/xfju8567 28966710 PMC5609662

[B21] Kavaliauskas M. Phillips S. M. (2016). Reliability and sensitivity of the 6 and 30 second Wingate tests in physically active males and females. Isokinet. Exercise Sci. 24, 277–284. doi: 10.3233/IES-160632

[B22] Krustrup P. Mohr M. Amstrup T. Rysgaard T. Johansen J. Steensberg A. . (2003). The yo-yo intermittent recovery test: Physiological response, reliability, and validity. Med. Sci. Sports Exercise 35, 697–705. doi: 10.1249/01.MSS.0000058441.94520.32 12673156

[B23] Linossier M. Denis C. Dormois D. Geyssant A. Lacour J. (2004). Ergometric and metabolic adaptation to a 5-s sprint training programme. Eur. J. Appl. Physiol. Occup. Physiol. 67, 408–414. doi: 10.1007/bf00376456 8299612

[B24] Naser N. Ali A. Macadam P. (2017). Physical and physiological demands of futsal. J. Exercise Sci. Fitness 15, 76–80. doi: 10.1016/j.jesf.2017.09.001 29541136 PMC5812879

[B25] Negra Y. Sammoud S. Ramirez-Campillo R. Bouguezzi R. Moran J. Chaabène H. (2023). The effects of repeated sprint training with vs. without change of direction on measures of physical fitness in youth male soccer players. J. Sports Med. Phys. Fitness 63 (1), 8–15. doi: 10.23736/S0022-4707.22.13521-8 35191289

[B26] Neves T. de A. Winckler C. Fernandes Guerra R. L. (2022). Effects of plyometric training combined with repeated sprints on physical performance in female handball athletes. Lecturas: Educación Física Y. Deportes 26 (286), 139–154. doi: 10.46642/efd.v26i286.3162

[B27] Soares-Caldeira L. F. de Souza E. A. de Freitas V. H. de Moraes S. M. Leicht A. S. Nakamura F. Y. (2014). Effects of additional repeated sprint training during preseason on performance, heart rate variability, and stress symptoms in futsal players: A randomized controlled trial. J. Strength Conditioning Res. 28, 2815–2826. doi: 10.1519/JSC.0000000000000461 24662230

[B28] Spencer M. Fitzsimons M. Dawson B. Bishop D. Goodman C. (2006). Reliability of a repeated-sprint test for field-hockey. J. Sci. Med. Sport 9, 181–184. doi: 10.1016/j.jsams.2005.05.001 16581293

[B29] Thurlow F. Huynh M. Townshend A. McLaren S. James L. Taylor J. . (2023). The effects of repeated-sprint training on physical fitness and physiological adaptation in athletes: A systematic review and meta-analysis. Sports Med. 54, 953–974. doi: 10.1007/s40279-023-01959-1 38041768

[B30] Tønnessen E. Shalfawi S. A. Haugen T. Enoksen E. (2011). The effect of 40-m repeated sprint training on maximum sprinting speed, repeated sprint speed endurance, vertical jump, and aerobic capacity in young elite male soccer players. J. Strength Conditioning Res. 25, 2364–2370. doi: 10.1519/JSC.0b013e3182023a65 21869624

[B31] Tortu E. Deliceoğlu G. (2024). Comparative analysis of energy system demands and performance metrics in professional soccer players: Running vs. cycling repeated sprint tests. Appl. Sci. 14 (15), 6518. doi: 10.3390/app14156518

[B32] Wang S. Tang J. Liu S. Li H. Li Q. Pan L. . (2025). Improving of 6 weeks of repeated sprint training on the aerobic and anaerobic power of college-age male rugby players. Front. Physiol. 16, 1620197. doi: 10.3389/fphys.2025.1620197 40800733 PMC12339488

[B33] Yuan Y. Soh K. G. Qi F. Bashir M. Zhao N. (2024). Effects of high-intensity interval training on selected indicators of physical fitness among male team-sport athletes: A systematic review and meta-analysis. PloS One 19, e0310955. doi: 10.1371/journal.pone.0310955 39536016 PMC11559996

[B34] Zhu D. Song D. Huang Z. (2025). Comparing individualized vs. non-individualized locomotor profiling on high-intensity interval training adaptations in soccer players: A randomized parallel study. J. Sports Sci. Med. 24, 503–512. doi: 10.52082/jssm.2025.503 40933332 PMC12418199

